# Generalized Linear Mixed Models for Changepoint Analysis of Biomedical Time Series Data

**DOI:** 10.1002/sim.70769

**Published:** 2026-10-13

**Authors:** Mark Fiecas, Rebecca Killick, Kathryn Cullen

**Affiliations:** ^1^ Division of Biostatistics and Health Data Science University of Minnesota Minneapolis Minnesota USA; ^2^ School of Mathematical Sciences Lancaster University Lancaster UK; ^3^ Department of Psychiatry and Behavioral Sciences University of Minnesota Minneapolis Minnesota USA

**Keywords:** covariance matrix, mean shift, mixed model, segmentation, time series

## Abstract

Changepoint analysis for time series data identifies the time points for which properties about the data shift. In this paper, we are motivated by two distinct types of biomedical time series data: digital health monitoring and neuroimaging. The neuroimaging study consists of multivariate time series obtained from resting‐state functional magnetic resonance imaging (fMRI). The digital health monitoring data consists of binary univariate time series observed within an individual throughout the day across multiple days of activity. The goal of the analysis is to model how physical activity changes within a day over a period of 56 days. The goal in the analysis of that data is to determine the association between dynamic functional connectivity (dFC), which is the interaction between different brain regions and how this interaction changes over time, and severity of non‐suicidal self‐injury (NSSI). In this paper, we leverage the framework of generalized linear mixed models to incorporate structure that is present in biomedical time series data for changepoint analysis. This structure could be, for example, the repeated measures of the time series data within an individual across multiple days or the interconnected community structure of brain networks. We embed the mixed model in a dynamic programming algorithm for detecting multiple changepoints. We evaluate the performance of our proposed method across several scenarios using simulations. Finally, we show the utility of our proposed method on our two distinct motivating applications.

## 1 Introduction

Technological innovations in the biomedical sciences have led to an increase in the amount of and the quality of time series data collected from individuals. In this paper, we consider two distinct examples of biomedical time series data: (1) data from at‐home devices in digital health, and (2) resting‐state functional resonance imaging (rs‐fMRI) data. In the former, we have activity monitoring data represented by a binary time series collected from a longitudinal study, and we are interested in modeling people's routine across 2 months and identify subtle departures from their routine. In the latter, rs‐fMRI data for an individual is a multivariate time series, and our goal is to quantify the changes in the strength of interactions between different regions of the brain, and investigate its utility as a biomarker for NSSI. In both examples, we are interested in identifying the times for which the properties of the time series data change, also known as changepoint analysis. The identification of these shifts and changes in the properties of the time series data provide important insights, for example, into the temporal dynamics of brain activity. Our main contribution in this article is to develop a generic framework for changepoint analysis of time series data. Though the two examples are very distinct, the same modeling framework can be adapted in a manner that leverages the structure present in the biomedical time series data.

Changepoint analysis has a long history and has been applied to time series data from many scientific fields, from climate time series to financial time series [1]. Generally, these methods were developed to identify changes in the mean, variance, or regression coefficients for continuous‐valued time series [2, 3, 4], and Poisson [5] and binomial time series data [6]. Methodological developments have allowed practitioners to use changepoint analysis across many scientific disciplines, from studying the climate [7], to patterns in human home activity [8], to brain connectivity [9, 10], and to closing prices of stock market indices [11], among many others. Biomedical time series data generally contain structure, which can arise from the underlying phenomenon of interest or from the study design. For instance, in our first example on the rs‐fMRI data, the time series are multivariate, and the different dimensions of the rs‐fMRI data form an interconnected community structure [12]. In the second example on the binary time series data collected from at‐home devices, the study had a longitudinal design, and so the time series data has two sources of correlation: the autocorrelation over time within a series and the within‐individual correlation from the repeated measures. Accounting for the structure in the time series data is critical to accurately characterize the phenomenon of interest, and will improve performance in estimation and statistical inference.

The mixed model framework is one approach to account for the structure present in the data through the careful specification of the random effects. For instance, for the rs‐fMRI data, the covariance structure of the random effects can model the interconnected community structure. Prior works have successfully used random effects in changepoint models to account for the longitudinal structure in the data [13, 14, 15]. These developments, however, were in the context of longitudinal models for the mean and not time series data. These developments nevertheless provide a foundation for using random effects for changepoint analysis, and so we will extend these methods to time series data. We will build on and extend the approach of using a mixed model framework for changepoint analysis for time series data.

The rest of this article is organized as follows. In Section 2, we describe the generalized linear mixed model, how to adapt the framework to multivariate time series, and how to embed this within the Pruned Exact Linear Time (PELT) algorithm for changepoint estimation. In Section 3, motivated by our two distinct examples, we use simulations to show the performance of our approach for both multivariate and binary time series data. In Section 4, we show the utility of our method in the analysis of the rs‐fMRI data and the at‐home activity monitoring data. We give our concluding thoughts in Section 5.

## 2 Methods

We consider a collection of time series $\left\{y_{t,i,j}\right\}$ observed at times t=1,…,n, for each group i=1,…,K where the *i*th group contains $k_{i}$ time series indexed as $j=1,\ldots ,k_{i}$. We use the term “group” in a generic manner, and we provide details later on the definition of a group. The total number of time series is $p=\sum_{i}k_{i}$. We assume there are M ordered changepoints at times $0=\tau_{0},\tau_{1},\ldots ,\tau_{M},\tau_{M+1}=n$ that partition the data into M+1 segments. We assume that the time series data within each segment follow a common distributional form but with parameters, particularly the covariance matrices or means, that differ between segments. In the following subsections, we leverage the mixed model framework for changepoint analysis of a univariate or multivariate time series. For the former, we are interested in mean shifts in the time series, and for the latter, we are interested in changes in the covariance matrix. In Sections 2.1 and 2.2, we describe the model assuming no changepoints, and then in Section 2.3, we describe how to embed these models within PELT for changepoint estimation.

### 2.1 Generalized Linear Mixed Models for Univariate Non‐Normal Time Series Data

We begin with a model for the mean of univariate time series. In our motivating example, we have a binary time series that marks movement or interaction with an object, and so we need a model for the probability of movement or interaction with an object. We therefore use the logit link function. Furthermore, the binary time series was collected across several days from several people, and so we need a model that borrows information across people while accounting for repeated measures within a person.

We consider the case where the mean of the data can be split into a global mean, μ, and a group‐specific deviation from the global mean, $\mu_{i},i=1,\ldots ,K$. The simplest form of a generalized linear mixed effects model (GLMER) is $g\left(E\left(y_{t,i,j}|\mu_{i}\right)\right)=\mu +\mu_{i},$ where g(⋅) is some link function. This is a general model applicable to data from the exponential family distributions. GLMERs are standard tools [16], and numerous packages (e.g., the lme4 package [17]) are available for estimation. We will embed the GLMER framework inside a changepoint algorithm (see Section 2.3) for changepoint estimation in the mean.

### 2.2 Linear Mixed Effects Covariance Model for Multivariate Time Series Data

We now consider the situation where we have multivariate time series data and are interested in the covariance matrix of the data. In our motivating example, we have rs‐fMRI data in the form of multivariate time series, and of interest is the correlation structure. The dimensions of the time series correspond to different subregions and regions of the brain, and can be grouped to form an interconnected community structure [12]. Thus, we need a model for the covariance matrix of the multivariate time series data that also accounts for the interconnected community structure. Consider the linear mixed model for the time series data $y_{t,i,j}=\mu_{i}+\epsilon_{t,i,j},$ where $\epsilon_{t,i,j}$ is iid normal across t,i, and j with mean zero and finite variance, and the random effects $\left\{\mu_{i}\right\}$ are normal and are mutually independent of the error terms $\left\{\epsilon_{t,i,j}\right\}$. The data is multivariate, where the indices i and j correspond to the dimensions of the time series. In our application, group i corresponds to a region of the brain with subregions indexed by $j=1,\ldots ,k_{i}$. Since we are interested primarily in changes in the p×p covariance matrix $\Sigma ={\left(\mathrm{Cov}\left(y_{t,i,j},y_{t,i^{\prime },j^{\prime }}\right)\right)}_{i,j,i^{\prime },j^{\prime }}$, we assume that the data have been centered so that the mean over time is the zero vector.

Let $y_{i,j}={\left(y_{1,i,j},\ldots ,y_{n,i,j}\right)}^{\prime }$ and let $y=\left(y_{1,1},y_{1,2},\ldots ,\right.y_{1,k_{1}},y_{2,1},\ldots y_{K,k_{K}}.$ be a $n\times \sum_{i}k_{i}$ matrix of our multivariate time series data. Let $\mu ={\left(\mu_{1},\ldots ,\mu_{K}\right)}^{\prime }$ with K×K covariance matrix $\Sigma_{\mu }=\mathrm{Cov}\left(\mu \right)={\left(\sigma_{\mu ,\mathrm{uv}}\right)}_{\mathrm{uv}}$ and $\epsilon ={\left(\epsilon_{11},\ldots ,\epsilon_{{\mathrm{Kk}}_{K}}\right)}^{\prime }$ with diagonal p×p covariance matrix $\Sigma_{\epsilon }=\mathrm{Cov}\left(\epsilon \right)=\mathrm{diag}\left(\sigma_{\epsilon ,11}^{2},\sigma_{\epsilon ,12}^{2},\ldots ,\sigma_{\epsilon ,1k_{1}}^{2},\sigma_{\epsilon ,21}^{2},\ldots ,\sigma_{\epsilon ,{\mathrm{Kk}}_{K}}^{2}\right)$. Then the covariance matrix of the data, Σ, is a function of $\Sigma_{\mu }$ and $\Sigma_{\epsilon }$. The assumptions we make about the within‐group and between‐group covariance structure lead to different structures for the covariance matrix of the data. We focus on two structures: (i) the Uniform‐Block structure (UB), and (ii) the Heterogeneous‐Block structure (HB).

The UB structure was previously developed, motivated by the strong interconnected community structure often present in high‐dimensional biomedical data [12]. The UB structure partitions the covariance matrix into submatrices such that the covariance matrix can be represented using a block Hadamard product. We need more notation to describe this representation. First, let $A=\mathrm{diag}\left(a_{1},\ldots ,a_{K}\right)$, where $a_{1},\ldots ,a_{K}>0$, and let $B=\left(b_{\mathrm{ij}}\right)$ be a K×K covariance matrix. Let ${\mathrm{Id}}_{u}$ be the u×u identity matrix, $1_{u\times v}$ be the u×v matrix of 1 s, and let $k=\left(k_{1},\ldots ,k_{K}\right)$ be a vector of positive integers that indicate the size of each group, assuming $k_{i}>1$ for each group i=1,…,K. Define $\mathrm{Id}\left(k\right)=\text{Bdiag}\left({\mathrm{Id}}_{k_{1}},\ldots ,{\mathrm{Id}}_{k_{K}}\right)$, where Bdiag(⋅) constructs a block‐diagonal matrix, define $J\left(k\right)=\left(1_{k_{u}\times k_{v}}\right)$, and let ∘ be the block Hadamard product such that $A\;\;\circ \;\;\mathrm{Id}\left(k\right)=\text{Bdiag}\left(a_{1}{\mathrm{Id}}_{k_{1}},\ldots ,a_{K}{\mathrm{Id}}_{k_{K}}\right)$ and $B\;\circ \;J\left(k\right)=\left(b_{\mathrm{uv}}1_{u\times v}\right)$. With these notations and definitions in place, then Σ has the UB structure if Σ=A∘Id(k)+B∘J(k). Relating this to the covariance matrices of the random effects, note that the UB structure imposes further assumptions on $\Sigma_{\epsilon }$. Specifically, suppose we have within‐group homogeneity, that is, $\sigma_{\epsilon ,i1}^{2}=\ldots =\sigma_{\epsilon ,{\mathrm{ik}}_{i}}^{2}$ for each i=1,…,K, and so we drop the last index to simplify the notation to $\sigma_{\epsilon ,i}^{2}$. This reduces the number of parameters of $\Sigma_{\epsilon }$ from p down to K, and we can instead use the representation ${\tilde{\Sigma }}_{\epsilon }=\mathrm{diag}\left(\sigma_{\epsilon ,1}^{2},\ldots ,\sigma_{\epsilon ,K}^{2}\right)$. From here, the linear mixed effects covariance model with the UB structure has the following form for the covariance matrix of the data: $\Sigma ={\tilde{\Sigma }}_{\epsilon }\;\circ \;\mathrm{Id}\left(k\right)+\Sigma_{\mu }\;\circ \;J\left(k\right)$.

Note that the UB structure corresponds to the assumption of homogeneity of the variance of the time series data across the time series within a group. The within‐group homogeneity may not be desirable, and one can instead keep $\Sigma_{\epsilon }$ to be a diagonal matrix with no further restrictions on the diagonal entries. This leads to the linear mixed effects covariance model with the Heterogeneous‐Block (HB) structure, which has the form $\Sigma =\Sigma_{\epsilon }+\Sigma_{\mu }\;\circ \;J\left(k\right).$ The HB structure still models the interconnected community structure like the UB structure, but allows for within‐group heterogeneity in the variance of the time series data. An important consideration, however, is the number of parameters. Note that ${\tilde{\Sigma }}_{\epsilon }\;\circ \;\mathrm{Id}\left(k\right)$ has K parameters, whereas $\Sigma_{\epsilon }$ has p parameters. Thus, the HB structure may be prohibitive for high‐dimensional time series, especially if the length of the time series is short relative to the number of dimensions. Altogether, the choice of the structure depends on the data, but can also be determined by, for example, exploratory data analyses on the diagonal entries of the covariance matrix within each group. Henceforth, we refer to the linear mixed effects covariance model using either the UB or HB structure as LMEC‐UB or LMEC‐HB, respectively. In Figure 1, we give a visual illustration on the block structure of Σ and how the entries of $\Sigma_{\mu }$ and $\Sigma_{\epsilon }$ contribute to the blocks.

**FIGURE 1:**
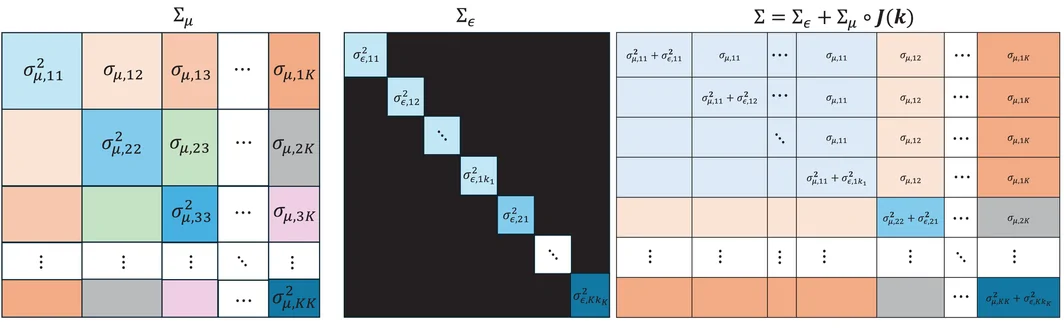
Visual structure of the covariance matrix for LMEC‐HB. $\Sigma_{\mu }$ is a K×K symmetric matrix, $\Sigma_{\epsilon }$ is a p×p diagonal matrix, Σ is a symmetric p×p matrix, and $J\left(k\right)=(1_{k_{u}\times k_{v})}.$ The colors correspond to the different blocks that make up the matrix defined by the groups. (Left) The diagonal entries correspond to the within‐group variability, and off‐diagonal entries correspond to the between‐group covariation. (Middle) Each entry along the diagonal corresponds to the deviation in variability from the corresponding group. For LMEC‐UB, the diagonal entries within a group are equal. Entries in black correspond to 0 s. (Right) The covariance matrix of the data is a function of both $\Sigma_{\mu }$, which contributes to both the diagonal and off‐diagonal entries, and $\Sigma_{\epsilon }$, which contributes only to the diagonal entries.

We now describe how to estimate the parameters of the model for both the UB and HB structures. We arrange the time series data column‐wise, keeping together the series that are in a group. Let S be the p×p sample covariance matrix for y. Our goal is to use the group structure in the data to provide an estimated matrix that has either a UB or HB structure. Note that a covariance matrix with either a UB or HB structure can be formed by block matrices with sizes determined by k. We use k in the same way to partition S to blocks so that $S=\left(S_{\mathrm{uv}}\right)$. If we use the UB structure, we have the following method‐of‐moments estimates:

(1) $${\hat{\sigma }}_{\mu ,\mathrm{uv}}=\left\{\begin{array}{ll} \frac{\mathrm{sum}\left(S_{\mathrm{uv}}\right)}{k_{u}k_{v}}, & u\neq v,\\ \frac{\mathrm{sum}\left(S_{\mathrm{uu}}\right)-\mathrm{tr}\left(S_{\mathrm{uu}}\right)}{k_{u}\left(k_{u}-1\right)}, & u=v, \end{array}\right.$$

(2) $${\hat{\sigma }}_{\epsilon ,u}^{2}=\frac{\mathrm{tr}\left(S_{\mathrm{uu}}\right)}{k_{u}}-{\hat{\sigma }}_{\mu ,\mathrm{uu}},$$

for u,v=1,…,K, where sum(⋅) denotes the sum of all the entries of the matrix, and tr(⋅) denotes the trace of the matrix [12]. These estimators have desirable theoretical properties, including strong consistency, asymptotic efficiency, and asymptotic normality [12].

Note that Equation (1) is the average of the diagonal elements (when u=v) or the average of the off‐diagonal elements (when u≠v) of $S_{\mathrm{uv}}$, and from Equation (2) we see that ${\hat{\sigma }}_{\mu ,\mathrm{uu}}+{\hat{\sigma }}_{\epsilon ,u}^{2}$ is the average of all the diagonal elements of $S_{\mathrm{uu}}$. We use this same logic if we instead use the HB structure, where we only need to revise the estimate of $\Sigma_{\epsilon }$. Let $S_{\mathrm{ij}}$ be the (i,j)th element of S. Then our estimate of the parameters for the HB structure is

(3) $${\hat{\sigma }}_{\epsilon ,\mathrm{uj}}^{2}=\mathrm{diag}{\left(S_{\mathrm{uu}}\right)}_{j}-{\hat{\sigma }}_{\mu ,\mathrm{uu}},$$

where $\mathrm{diag}{\left(S_{\mathrm{uu}}\right)}_{j}$ is the *j*th diagonal element of $S_{\mathrm{uu}}$.

### 2.3 Changepoint Estimation Using PELT

We will embed the models we described in Sections 2.1 and 2.2 inside a changepoint algorithm called PELT, which we describe in this section. For the GLMER, changepoints correspond to a changes in the mean, and for the LMEC, changepoints correspond to changes in the covariance matrix. Recall that the changepoints are at times $0=\tau_{0},\tau_{1},\ldots ,\tau_{M},\tau_{M+1}=n$. With these changepoints, the model parameters in segment m, defined by the time interval $\left(\tau_{m},\tau_{m+1}\right]$, are assumed to be the same, but the model parameters can vary between segments.

Let $y_{t}=\left(y_{t,1,1},\ldots ,y_{t,1,k_{1}},y_{t,2,1},\ldots ,y_{t,K,k_{K}}\right)$ be our time series data indexed by time, collected within and between groups. Optimizing a measure of fit of the mixed effects models described in the previous sections is equivalent to optimizing the no changepoint scenario $\sum_{s=1}^{n}C\left(y_{s}|\theta \right)$ for θ. Here C(⋅) is a measure of fit which, throughout this paper, will be twice the negative log‐likelihood. As written, optimizing $\sum_{s=1}^{n}C\left(y_{s}|\theta \right)$ fits a single model to obtain one set of parameters for all time points. However, the parameters can change between the segments defined by the changepoints, but both the number of changepoints and their locations are not known. Thus, our goal is to optimize

(4) $$\min_{\tau ,M}\;\sum_{m=0}^{M}\;\sum_{s=\tau_{m}+1}^{\tau_{m+1}}C\left(y_{s}|{\hat{\theta }}_{m}\right),$$

given parameter estimates $\left\{{\hat{\theta }}_{m}\right\}$. Due to the discrete nature of both the number and location of changepoints, standard estimation methods cannot be directly applied. We now have a model selection problem where we need to select the appropriate number of changepoints and, in turn, their location. This is akin to choosing the number of regressors in a regression problem. Left free, the optimization of Equation (4) would choose the maximum number of changepoints and so, as in the regression context, we need to penalize to obtain a parsimonious fit. Penalties of the form CMlog(n) are consistent for likelihood‐based cost functions C(⋅) and constant C with respect to n [18]. We revise Equation (4), and now our goal is to optimize

(5) $$F\left(n\right)=\min_{\tau ,M}\;\sum_{m=0}^{M}\;\sum_{s=\tau_{m}+1}^{\tau_{m+1}}C\left(y_{s}|{\hat{\theta }}_{m}\right)+\mathrm{CM}\log\left(n\right).$$

Optimizing Equation (5) over all possible combinations of M and τ is a computationally intensive task. A combination of dynamic programming and pruning of the search space can reduce the computational burden from $O\left(2^{n}\right)$ to O(n) [2]. Due to the independence of the segments, dynamic programming allows us to rewrite the search for all changepoints in (5) in terms of the search for the last changepoint,

(6) $$F\left(n\right)=\min_{\tau^{*}}\;F\left(\tau^{*}\right)+\sum_{s=\tau^{*}}^{n}C\left(y_{s}|{\hat{\theta }}_{M}\right)+C\log\left(n\right).$$

Computing $F\left(\tau^{*}\right)$ recursively for $\tau^{*}=1,\ldots ,n$ recovers the optimal set of changepoints for penalty CMlog(n) in $O\left(n^{2}\right)$ computational time. To reduce this to O(n) one can prune the minimization in Equation (6). As the minimization is looking for the best last changepoint location, where there is an obvious changepoint location, the best last changepoint is unlikely to be prior to the obvious changepoint. This intuition leads to a mathematically optimal pruning rule for an individual $\tau^{*}$, if it satisfies $F\left(\tau^{*}\right)+\sum_{s=\tau^{*}}^{t}C\left(y_{s}|{\hat{\theta }}_{M}\right)\geq F\left(t\right).$ Intuitively this says that if at any time in the recursive computation, a candidate last changepoint location, $\tau^{*}$, is more than Clog(n) larger than the optimal likelihood at that time, t, it can never be the last changepoint at any future point so it can be pruned from the minimization in (6). This pruning rule exactly solves the same minimization problem without approximation [2]. This algorithm is called PELT, for Pruned Exact Linear Time [2].

We use PELT as a wrapper for each of GLMER, LMEC‐UB, and LMEC‐HB discussed earlier and using the negative twice the log‐likelihood as C(⋅) in (6), giving rise to PELT‐GLMER, PELT‐LMEC‐UB, and PELT‐LMEC‐HB, respectively. For multivariate time series, when referring to the general modeling framework without specifying the UB or HB structure, we use the term PELT‐LMEC. The computational cost of these methods is O(Ln) where L is the computational order of evaluating the likelihood for a single segment.

### 2.4 Practical Considerations

Our proposed method has three practical concerns: (i) choice of parameters, (ii) the grouping structure, and (iii) model assumptions.

The minimum segment length balances the need for having enough data to estimate the model parameters (regression or covariance matrix) with being localized enough in time. Consequently, our method may miss changepoints that occur rapidly. For PELT‐GLMER, we use a minimum segment length of p. For the covariance model, a recently‐developed method [19] used a minimum segment length of 4p, but from our explorations of the impact of this parameter on the performance of the method, we recommend a smaller value of 2p for the minimum segment length. Note that PELT‐LMEC‐UB has $K+\left(\begin{array}{c} K+1\\ 2 \end{array}\right)$ parameters to estimate whereas PELT‐LMEC‐HB has $p+\left(\begin{array}{c} K+1\\ 2 \end{array}\right)$ parameters. Assuming that K is much smaller than p, the value of 2p for the minimum segment length provides a sufficient amount of data for estimating the parameters for PELT‐LMEC‐UB or PELT‐LMEC‐HB.

Recall the penalty term in PELT that guarantees asymptotic consistency [18] is CMlog(n). Another parameter to set is the constant C in this penalty term. If C is too small, then there will be more candidate changepoints, thereby increasing the possibility for falsely detected changepoint locations. If C is too large, then only changepoints that lead to very large changes in the likelihood will be detected. For all methods, we use a BIC‐like penalty; C=p for PELT‐GLMER and $C=K+\left(\begin{array}{c} K+1\\ 2 \end{array}\right)$ for PELT‐LMEC‐UB and PELT‐LMEC‐HB, which is also the number of parameters to estimate in the covariance matrix for LMEC‐UB. In investigations not shown here, setting $C=p+\left(\begin{array}{c} K+1\\ 2 \end{array}\right)$, which is the number of parameters for LMEC‐HB, led to unsatisfactory performance for changepoint detection, especially whenever p was relatively large. It should be noted that the CMlog(n) form is under model assumptions. If these are violated, for example heavier tails, outliers, or autocorrelation, then this penalty should be modified. For heavier tails and positive autocorrelation, the penalty needs to be increased to account for this. For negative autocorrelation, the penalty needs to be decrease. For outliers, these can either be pre or post‐processed (an additive outlier will result in a segment equal to the minimum segment length) or the cost function can be modified to a more robust version, for example, Huber loss [20]. There are other ways that penalties can be chosen, including cross‐validation [21], semi‐supervised learning [22], and elbow‐plots [23].

Our proposed method assumes a fixed and known grouping structure for the time series data. In our application for PELT‐GLMER, the grouping structure was induced by the longitudinal design. However, in our application for PELT‐LMEC, we used domain knowledge about the study and the data to create the groups. Misspecification of the groups, however, can affect the estimates of the blocks in each of the UB and HB structures, thereby affecting model fit. One could use a community detection or clustering algorithm to identify the groups in a data‐driven manner [24]. We show in our simulation study the impact of misspecified groups on the performance.

Time series data often exhibit temporal autocorrelation. However, our proposed method using our recommended penalty term assumes no temporal autocorrelation. Prior works showed that temporal autocorrelation leads to an overestimation of the number of changepoints [3, 19, 25]. A recent work [25] showed how one can incorporate the autocorrelation structure of the data for changepoint detection in univariate time series, but adapting this to multivariate time series is beyond the scope of this work. As we will see in our simulations, autocorrelation in the multivariate time series data can negatively impact the accuracy of changepoint estimation. To satisfy the assumption of no temporal autocorrelation, we pre‐whitened the time series.

## 3 Simulation Study

In this section we will assess the different base random effects models via simulation studies inspired by our applications in digital health. The goal of our simulations is to show when these approaches perform well and the impact of violations of assumptions that may occur in applications.

We give a quantitative assessment of performance using two metrics: (i) the number of detected changepoints ($|M-\hat{M}|$), and (ii) the mean absolute error (MAE), defined as $\mathrm{MAE}=\frac{1}{n}\sum_{t=1}^{n}{\left\|{\hat{\theta }}_{t}-\theta_{t}\right\|}_{1},$ where θ is the set of estimated parameters, for example, covariance matrix. The MAE assesses the impact of the estimated changepoints on the estimates of the parameters across all time points and should be viewed in conjunction with the number and location of changepoints. For changepoint locations, we give a qualitative visual assessment. For some applications accurately estimating the number and location of changepoints is the primary objective, for others, the parameter estimation is paramount and the changepoints are a nuisance.

### 3.1 PELT‐GLMER

The settings we use is motivated by our digital health monitoring data. We simulate Bernoulli data from K=4 groups (people), each time series represents either a day (n=96) or a week (n=672), and we have 14 replicates (2 weeks of days and approximately a quarter for weekly data). We set the global mean probability to be either constant or to vary at changepoints, and for each replicate we independently simulated the global mean from a Uniform (0.1, 0.9) distribution. Similarly, we set the group effect to be either constant or vary at changepoints with the added scenario where a single group or all groups can change at each changepoint. We simulated the group effect independently from a Normal distribution with mean 0 and standard deviation 0.05. When simulating the data, we curtailed the sum of the group and global effect to be between 0.01 and 0.99. We simulated the shorter daily series with three changepoints at 28, 34 and 88. We simulated the longer weekly series with 14 changepoints at 27, 87, 121, 181, 222, 282, 316, 376, 410, 470, 511, 571, 605, and 665. These changepoints represent wake and sleep with some variability across days. We repeated each scenario 500 times. Note that this random effects design for the simulation parameters means that for each of the 500 scenarios, for each changepoint, the effect size will be random. The maximum change size will be 0.8 (from 0.1 to 0.9 or vice versa) and the minimum change size will be close to 0. Not all changes will be statistically detectable. This gives us the ability to generalize to the population of effects (the entire sample space).

We compare PELTM‐GLMER with two approaches that both ignore the group structure: (i) the Binomial likelihood which pools the observations across replications and groups to detect a change in the Binomial probability of events (PELT‐Bin), and (ii) the Bernoulli likelihood which treats each time series as an independent realization of a Bernoulli process (PELT‐Bern). We expect the Bernoulli approach to be the least powerful as it has 96 observations to detect a change. The Binomial approach pools information across replicates and groups to boost power but ignores the group structure, and so alternate changes (decrease in one group, increase in another) are masked.

Figure 2 shows that when the global mean changes, there is little effect whether the group effects also change. PELT‐Bin and PELT‐GLMER accurately detected the changepoint locations, with the latter having much better power. On the other hand, while the distribution of the detected changepoints by PELT‐Bern correctly had modes at the true changepoint locations, these estimates were not precise. In contrast, Figure 3 demonstrates that when the global mean stays constant, the no group change is the null model, and so no changepoints should be detected. PELT‐Bin had very little changes, and PELT‐GLMER successfully detected no changes. PELT‐Bern, however, falsely detected many changepoints. When the groups truly had changes, then the detection frequency for both PELT‐Bin and PELT‐GLMER increased, withPELT‐GLMER yielding more accurate estimates of the changepoint locations. In results not shown here, with longer time series, the performances of the methods relative to each other are the same, but the precision of the estimates of the changepoint locations are better.

**FIGURE 2:**
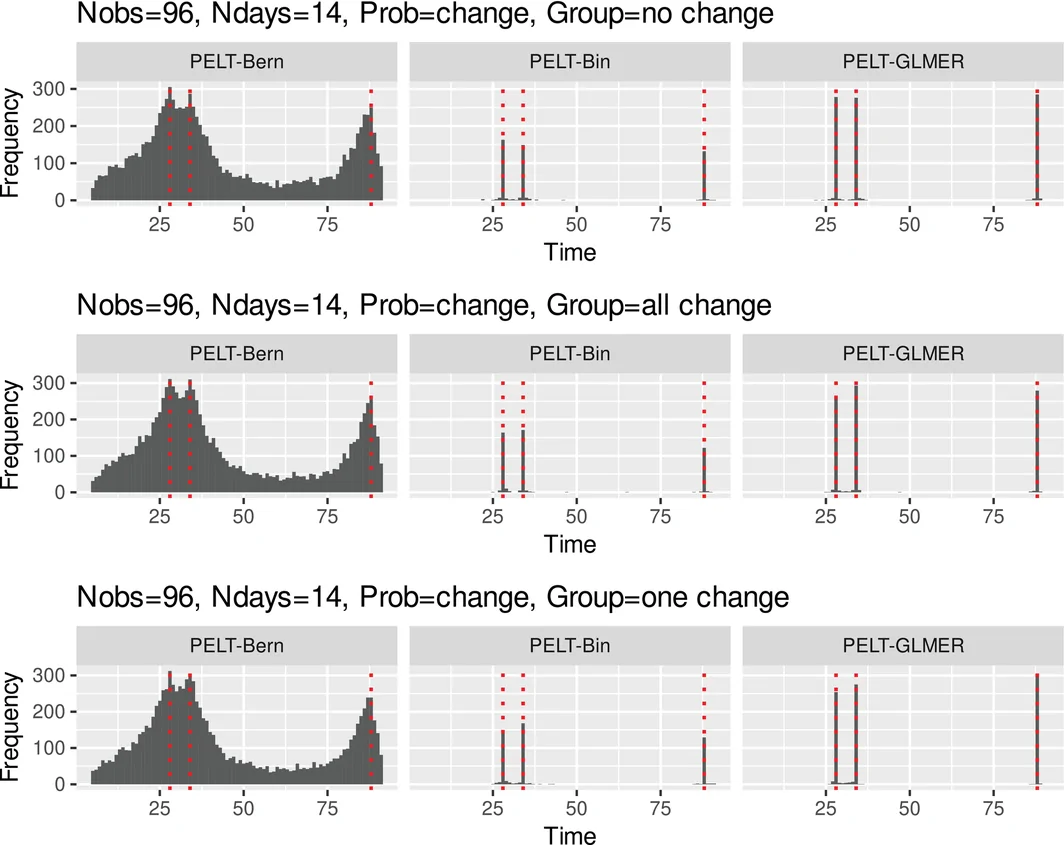
Histogram of the location of the detected changepoints in the simulations from n=96 observations, with 14 replicates, where the global mean probability changes. Dotted red lines correspond to the true changepoint locations. (Top) No group structure changes. (Middle) All group structures change. (Bottom) One group structure changes.

**FIGURE 3:**
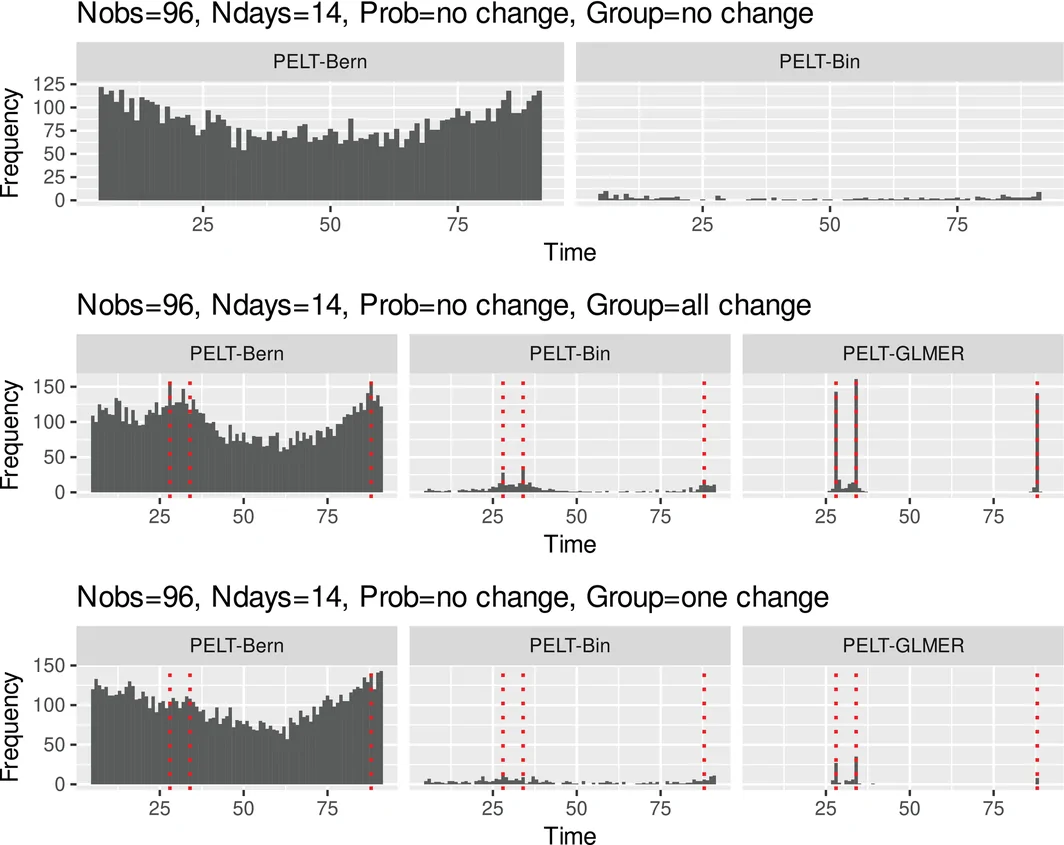
Histogram of the location of the detected changepoints in the simulations from n=96 observations, with 14 replicates, where the global mean probability does not change. Dotted red lines correspond to the true changepoint locations. (Top) No group structure changes. Note that there is no PELT‐GLMER histogram as no replicates identify changepoints. (Middle) All group structures change. (Bottom) One group structure changes.

Comparing across methods, we see that in all cases, the Bernoulli results have more variability. Note that this is because we are performing 500×4×14 independent analyses that we pool naively here. This means that if one time series detects changes at (27, 34) and another at (28, 35) this is recorded as a final set of changes at (27, 28, 34, 35). This favors the Bernoulli approach in terms of recording the frequency of the correct changepoint locations (as it gets 56 tries to find the correct value, compared to 1 for the other methods). The cost for this is in the variability in the location of the changepoint estimates. While there may be alternative ways of pooling across time series, that is not the interest of this paper. Turning to the Binomial, the changepoints are at similar locations and with similar variability to the GLMER but with lower frequency as it does not take advantage of the grouping structure. This is particularly pronounced in Figure 3.

In Table 1, we show the performance at estimating the number of changepoints. PELT‐Bern had a very large error rate due to the high number of false detections that we saw in Figures 2 and 3. PELT‐GLMER generally had superior performance at estimating the number of changepoints.

**TABLE 1:** $|M-\hat{M}|$ for the estimated number of changepoints across all scenarios.

Simulation setting			$|M-\hat{M}|$		
n	Global	Group	PELT‐Bern	PELT‐Bin	PELT‐GLMER
96	Y	N	17.650	2.004	**1.232**
	Y	All	17.734	2.004	**1.246**
	Y	1	17.386	2.000	**1.258**
672	Y	N	21.954	2.000	**0.670**
	Y	All	24.108	2.000	**0.508**
	Y	1	22.478	2.000	**0.600**
96	N	N	16.373	2.642	**0**
	N	All	16.518	2.256	**1.950**
	N	1	14.402	**2.434**	2.812
672	N	N	13.784	2.770	**0**
	N	All	26.096	2.082	**0.425**
	N	1	14.448	**2.248**	2.280

*Note:* The method that performed the best at a given simulation setting combination has its entry in boldface font.

### 3.2 PELT‐LMEC

We now turn to using PELT‐LMEC for changepoint estimation for multivariate time series. We used six scenarios for our simulation study:

1. **Zero changepoints**. We simulated data from a zero‐mean multivariate normal distribution with random covariance matrices. This scenario assesses the false positive rate for each method.
2. **One changepoint**. We set the changepoint at $\tau_{1}=n/2$, and within each segment, we simulated data from a zero‐mean multivariate normal distribution with random covariance matrices.
3. **Multiple changepoints**. We set the changepoints at $\tau_{1}=0.4n,\tau_{2}=0.6n$, and $\tau_{3}=0.75n$. Within each segment, we similarly simulated data from a zero‐mean multivariate normal distribution with random covariance matrices.
4. **Multiple changepoints, misspecified groups**. We follow the same setup as in Scenario 3. However, we deliberately misspecified too few groups to assess the impact of group misspecification.
5. **Multiple changepoints, autocorrelated data**. We follow the same setup as in Scenario 3. However, within each segment, we set $y_{i,j,t}=\phi y_{i,j,t-1}+z_{i,j,t}$, where, for a given t, we simulated $z_{t}$ from a multivariate normal distribution with random covariance matrices. Note that our method assumes temporal independence, thus, this scenario assesses of the impact of temporal autocorrelation.
6. **One changepoint, fixed** $\Sigma_{\mu }$. We follow the same setup as in Scenario 2. However, between segments, $\Sigma_{\mu }$ is kept fixed.

For Scenario 4, a “misspecified group” refers to a subregion of the brain (or a parcel) that was misclassified to belong to a region. In each of the above scenarios, we considered n∈{500,1000} and K=4 groups such that all groups contain $k_{i}=5$ or $k_{i}=15$ many time series. Thus, the dimensions for both Σ and $\Sigma_{\varepsilon }$ was either 20×20 or 60×60. For Scenario 4, the dimensions for $\Sigma_{\mu }$ was 5×5, but for the other scenarios its dimensions were 4×4. Note for Scenario 4 that this implies that there were truly 5 groups, but in the model we set K=4. Excluding Scenario 6, to construct $\Sigma_{\mu }$ for each segment, we first simulated a random matrix from the Wishart distribution with degrees of freedom set to the number of dimensions of the data (i.e., either 20 or 60) with scale matrix the identity matrix of appropriate dimension, and then converted the simulated matrix to a correlation matrix. For Scenario 6, we simulated $\Sigma_{\mu }$ as above, but we keep it fixed across segments. To construct $\Sigma_{\epsilon }$ for each segment, we created a diagonal matrix with each diagonal entry simulated from the Uniform (0.75,1.25) distribution. For Scenario 5 with autocorrelated data, we used the above for simulating a structured covariance matrix to create the covariance matrix for $z_{t}$, and we set ϕ=0.6.

We compare the performance of each of PELT‐LMEC‐UB and PELT‐LMEC‐HB in each of the above scenarios to two competing methods: the Ratio method [19] and the Wild Sparsified Binary Segmentation (BSCOV) [26], which we now briefly describe. The Ratio method uses a test statistic based on the eigenvalues of the covariance matrices calculated between two segments of the data. The Ratio method uses binary segmentation for changepoint detection. BSCOV, on the other hand, fits an approximate factor model to the multivariate time series data and then uses wild binary segmentation for identifying the changepoints in the factor components.

In Tables 2 and 3, we see how each method performs in estimating the number of changepoints and the covariance matrices. In Scenario 1, all methods performed very well with respect to their false positives. For the MAE in Table 3, we see that PELT‐LMEC‐HB and PELT‐LMEC‐UB both performed better than Ratio, and this is due to PELT‐LMEC‐HB and PELT‐LMEC‐UB assuming a structure for the covariance matrix, whereas Ratio was unstructured. Interestingly, even though the true covariance matrices were heterogeneous along the diagonal entries, assuming a UB structure led to better performance with respect to MAE even at the higher dimension, and this is likely due to the lower variance in the estimates as a result of averaging the estimates of the diagonal entries within a block.

**TABLE 2:** $|M-\hat{M}|$ for the estimated covariance matrix across all scenarios.

Simulation setting			$|M-\hat{M}|$			
			PELT		PELT	
	p	n	BSCOV	LMEC‐HB	LMEC‐UB	Ratio
M=0	20	500	**0.00**	**0.00**	**0.00**	0.02
		1000	**0.00**	**0.00**	**0.00**	**0.00**
	60	500	**0.00**	**0.00**	**0.00**	**0.00**
		1000	**0.00**	**0.00**	**0.00**	**0.00**
M=1	20	500	0.68	**0.00**	0.50	0.04
		1000	0.40	**0.00**	0.15	0.01
	60	500	0.93	**0.00**	0.69	0.04
		1000	0.89	**0.00**	0.31	**0.00**
M=3	20	500	2.76	**2.53**	2.80	2.72
		1000	2.51	**1.54**	2.20	2.08
	60	500	2.93	**2.45**	3.00	3.00
		1000	2.93	**1.06**	2.90	2.96
M=3, misspecified groups	20	500	2.65	2.55	2.81	**2.52**
		1000	2.31	1.71	2.24	**1.54**
	60	500	2.92	**1.73**	2.94	3.00
		1000	2.90	**0.60**	2.69	2.95
M=3, autocorrelation	20	500	**1.96**	2.33	2.44	2.16
		1000	2.37	**1.45**	1.73	8.38
	60	500	2.68	2.81	2.87	**2.00**
		1000	2.75	2.07	2.62	**0.16**
M=1, fixed $\Sigma_{\mu }$	20	500	0.99	**0.95**	1.00	0.96
		1000	1.00	**0.61**	1.00	0.85
	60	500	1.00	**0.26**	1.00	1.00
		1000	1.00	**0.00**	0.99	0.88

*Note:* The method that performed the best at a given simulation setting and (p,n) combination had its entry in boldface font.

**TABLE 3:** Mean absolute error (MAE) for the estimated covariance matrix across all scenarios.

			MAE		
			PELT	PELT	
Simulation setting	p	n	LMEC‐HB	LMEC‐UB	Ratio
M=0	20	500	443.67	**419.37**	800.86
		1000	442.03	**418.22**	795.80
	60	500	3079.58	**2989.64**	7049.65
		1000	3008.23	**2914.25**	6986.55
M=1	20	500	465.24	**440.36**	816.96
		1000	450.39	**431.95**	813.02
	60	500	**3071.74**	3084.37	7140.78
		1000	3111.78	**3073.81**	7093.82
M=3	20	500	379.61	**366.79**	555.48
		1000	383.31	**369.59**	552.91
	60	500	2914.51	**2813.91**	4823.67
		1000	2971.55	**2820.12**	4859.30
M=3, misspecified groups	20	500	291.98	**276.67**	558.65
		1000	292.60	**276.30**	556.11
	60	500	2093.32	**2000.45**	4787.28
		1000	2136.93	**2023.94**	4853.28
M=3, autocorrelation	20	500	547.18	**521.74**	827.61
		1000	543.32	**528.35**	826.35
	60	500	3972.77	**3779.38**	6912.02
		1000	4142.04	**3936.08**	7172.31
M=1, fixed $\Sigma_{\mu }$	20	500	373.97	**361.88**	555.05
		1000	363.54	**347.63**	539.91
	60	500	2850.35	**2745.03**	4818.49
		1000	2881.80	**2800.65**	4882.57

*Note:* Since BSCOV fits a factor model to the data and does not explicitly estimate covariance matrices, we did not evaluate its MAE for estimating the covariance matrices, and hence was omitted. The method that performed the best at a given simulation setting and (p,n) combination had its entry in boldface font.

We now describe the results when there was M=1 changepoint. In Scenario 2, PELT‐LMEC‐HB performed the best at estimating the single changepoint. The Ratio method also performed very well, and BSCOV performed the worst of the four methods. We also see that as the sample size increased each method performed better at estimating the number of changepoints. From Figure 4a, we see that, for p=20 and n=1000, the location of the changepoints, if a method detected one, was at or very close to the true changepoint location. In Scenario 6, the change in the covariance structure is more subtle, and we can see that all methods struggled especially for p=20 and n=500. However, PELT‐LMEC‐HB outperformed all other methods.

**FIGURE 4:**
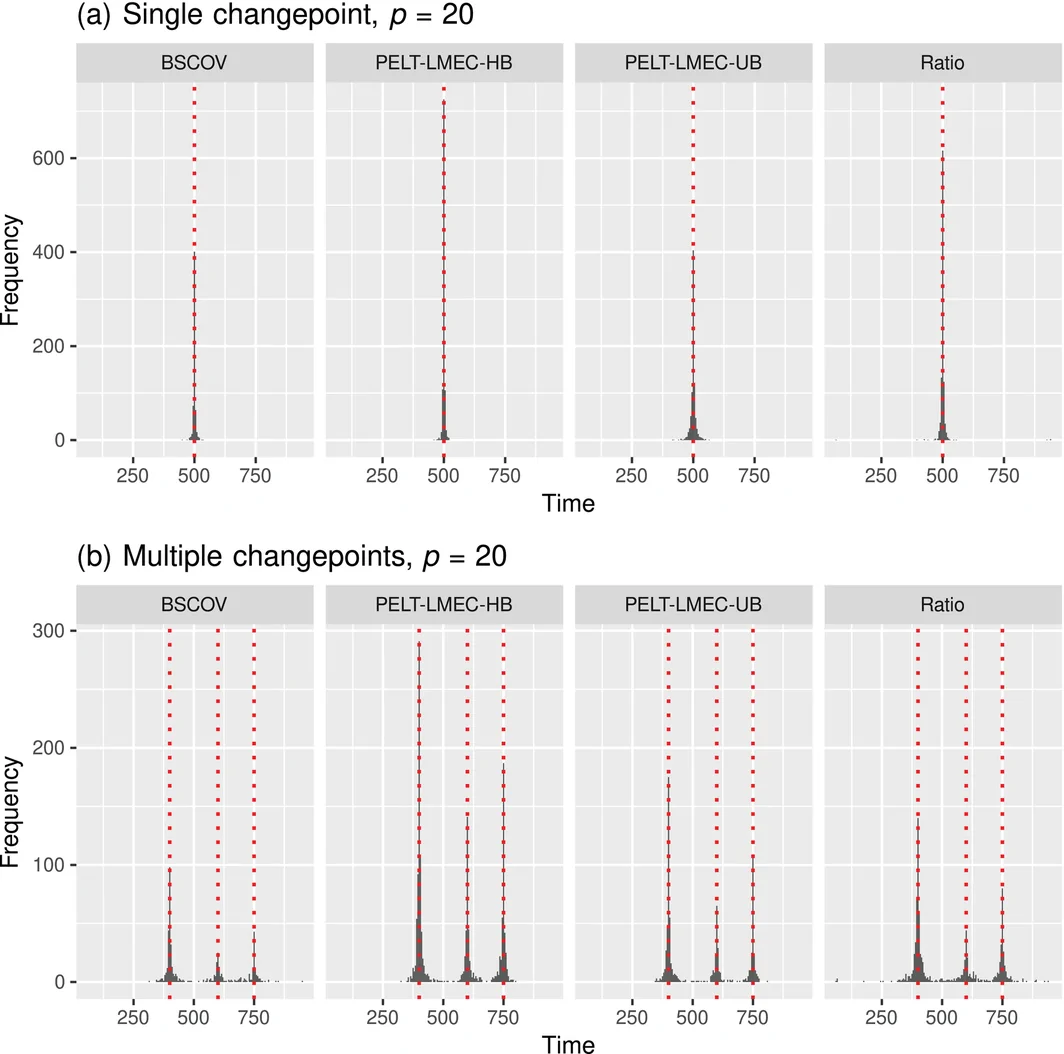
Histogram of the location of the detected changepoints in the simulations from two scenarios obtained from each of the four methods. Dotted red lines correspond to the true changepoint locations. (a) Single changepoint, with p=20 and n=1000. (b) Multiple changepoints, with p=20 and n=1000.

In Scenario 3, all methods struggled at detecting all three changepoints, especially when p=60. Recall that $\tau_{2}=0.6n$ and $\tau_{3}=0.75n$, and so since they were relatively close in time it was difficult for the methods to detect both of these changepoints. Indeed, from Figure 4b we see that $\tau_{1}$ was the easiest changepoint for all methods to detect since that changepoint had the greatest frequency of being detected relative to the other two changepoints. However, when p=60 and n=500, all methods struggled to detect any of the changepoints. For p=60 and n=1000, PELT‐LMEC‐HB performed the best since it usually detected at least two of the changepoints, with $\tau_{2}$ having the lowest frequency of being detected as shown in Figure 5b. However, the other methods continued to struggle to detect any of the changepoints. Indeed, from Figure 5a, we see the poor performances of all methods except PELT‐LMEC‐HB at detecting the changepoints. Note, however, that PELT‐LMEC‐UB generally had lower MAEs than PELT‐LMEC‐HB as shown in Table 3, so even though the uniform assumption led to better estimates of the covariance matrices, the heterogeneity assumption led to better estimates of the number and location of changepoints.

**FIGURE 5:**
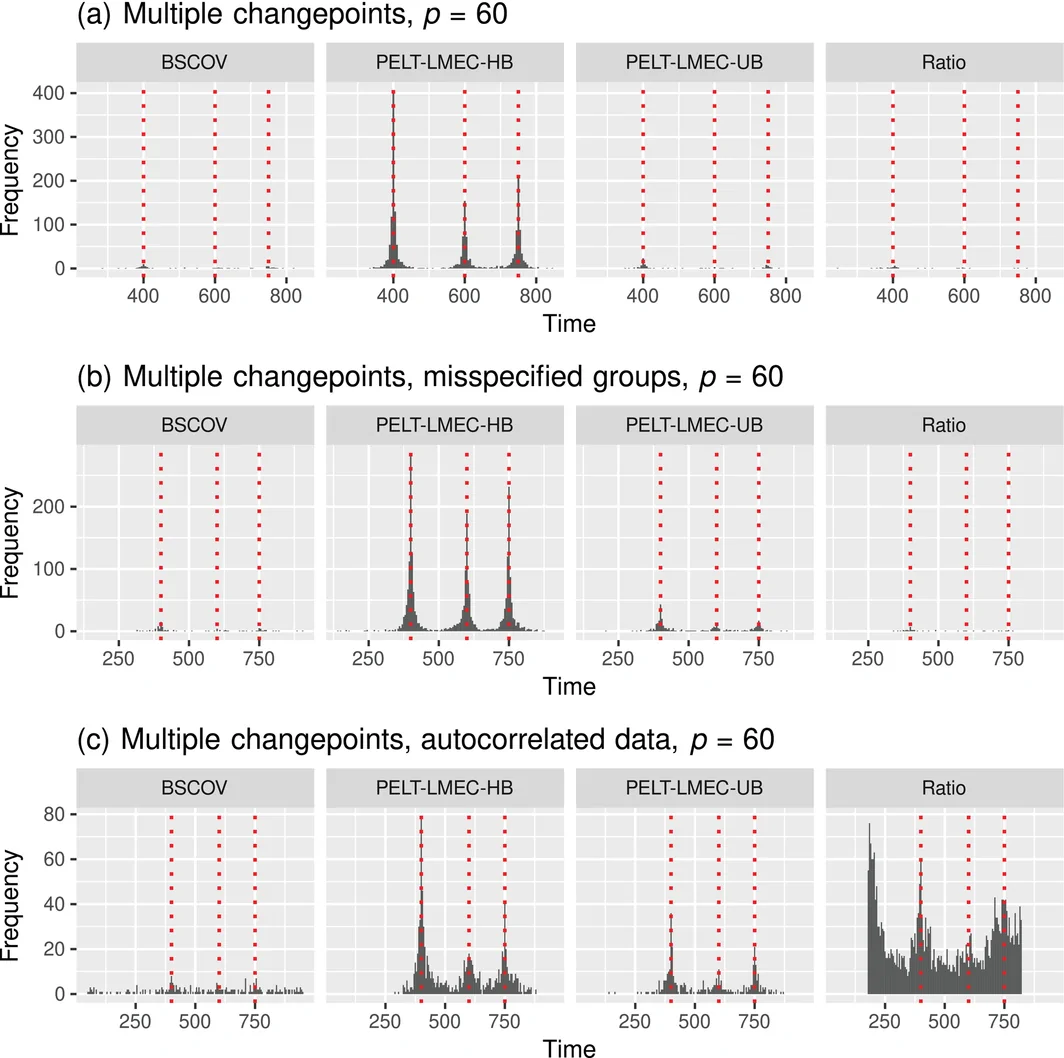
Histogram of the location of the detected changepoints in the simulations from two scenarios obtained from each of the four methods. Dotted red lines correspond to the true changepoint locations. (a) Multiple changepoints, with p=60 and n=1000. (b) Multiple changepoints and misspecified groups, with p=60 and n=1000. (c) Multiple changepoints and autocorrelated data, with p=60 and n=1000.

In Scenario 4, the MAEs were smaller than those in Scenario 3. This is likely due to the greater amount of averaging across the dimensions of the time series, leading to a decrease in the variance of the estimates that overcame the increase in the bias. While it may also seem that both PELT‐LMEC‐HB and PELT‐LMEC‐UB better detected the number of changepoints, we point out that, from a visual assessment of Figure 5, the precision of the estimates of the changepoint locations appears to be lower, since the distribution of the estimated changepoints appears to be wider around the true changepoint locations.

Autocorrelation generally inflates false positives in changepoint detection methods that were not explicitly designed to account for autocorrelation [3, 19, 25], and we observe that phenomenon in Scenario 5. In Figure 5b, we see that all methods are not as precise at estimating the changepoint locations. While Table 2 suggests that the Ratio method can estimate very well the number of changepoints for p=60 and n=1000, we see in Figure 5b that the location of these changepoints are not temporally precise at the true changepoint locations, with the most common changepoint it detected not anywhere close to the location of a true changepoint. PELT‐LMEC‐HB and PELT‐LMEC‐UB both better estimate the changepoint locations relative to BSCOV and Ratio, but the precision of the estimates were also negatively impacted by the presence of autocorrelation. This motivates the need for removing the autocorrelation prior to analyses.

## 4 Applications

### 4.1 At‐Home Digital Health Monitoring

Traditionally, point of contact with healthcare professionals was the way clinicians could monitor someone's health clinically. Over time, this leads to increased stays in hospital, inconvenient repeated appointments, or discharge followed by adverse events in order to monitor effectively. With the advent of digital health monitoring, a patient can permit healthcare providers to receive updates from tested and approved devices at home. Similarly, those interested in monitoring their long‐term health can purchase devices that track health metrics over time. Many of these devices are wrist‐worn for continuous measurement or reminders are given through apps to take measurements. These devices often have low adherence in specific patient groups. An alternative approach is passive monitoring within the home. These devices provide a different type of data which can often be combined with other health measurements when available.

We analyze data from four people who use the Howz at‐home passive activity monitoring. The passive sensors record movement or interaction with an object, for example, opening the fridge or turning the kettle on. Howz summarizes the data as a yes/no for whether there is any activity within a 15‐min window. This results in 96 yes/no observations per day and we analyze 56 days of activity for each person.

Utilizing the method described in Section 2.1, we fit PELT‐GLMER using a Bernoulli distribution with logit link function to each day of data. Each day of observations from an individual is a separate time series within the same group; thus, we have four groups. Prior analyses of similar Howz data [8, 27] showed that individuals in this cohort tended to have similar behavior across days. Thus, we expect to see global effects, such as similar wake‐up and bedtime changes, in activity levels alongside group effects related to an individual's routine. Being able to describe routines that are common across individuals as well as individual‐level behavior is important in providing insights for different purposes.

We ran PELT‐GLMER with a penalty of 56log(96) for 56 days of data each with 96 observations. Figure 6 shows the data with the changepoints overlayed. It is clear that the first changepoint (7 a.m.) is driven by a change in the first two participants. The second change (8:30 a.m.) looks to be driven by participants 1, 3, and 4. The 5:30 p.m. change is driven by Participant 3 increasing, and Participant 4 decreasing activity. The final change at 10:15 p.m. looks to be bedtime for Participants 2 and 4, and the start of a bedtime routine (increase in activity) for Participant 1. Table 4 depicts the global mean probability of activity for each segment along with their associated bootstrap 95% confidence intervals. This reflects the fact that the overall probability of activity is higher during the day than in the two nighttime periods, and increases as the day progresses. Table 5 then depicts the deviation, on the logit scale, of the groups (each person) from this average behavior. This correlates with what we can determine visually, showing the largest variation across individuals is in the initial nighttime (00:00–07:30) period corresponding to individuals who conduct activity (e.g., visiting the bathroom) over night frequently to not at all.

**FIGURE 6:**
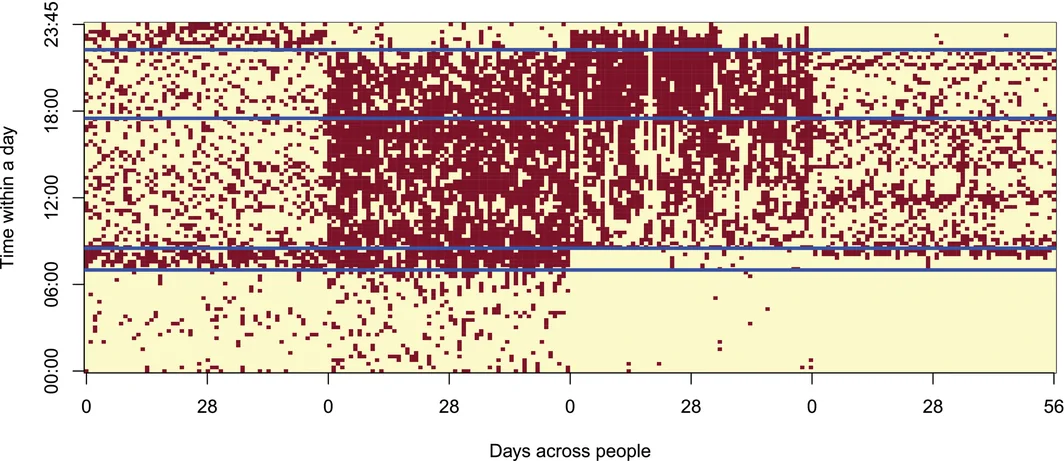
Daily Howz data with changepoints at 7 a.m., 8:30 a.m., 5:30 p.m., and 10:15 p.m. The x‐axis shows days for each person, across four people.

**TABLE 4:** Probability of activity and their associated 95% bootstrap confidence intervals in the Howz daily analysis.

Segment	1	2	3	4	5
2.5% level	0.000	0.109	0.278	0.229	0.024
Mean	0.011	0.367	0.483	0.497	0.141
97.5% level	0.176	0.732	0.698	0.752	0.501

**TABLE 5:** Contribution of the random effects, on the logit scale, on the probability of activity across segments and people in the Howz daily analysis.

	Person			
Segment	1	2	3	4
1	8.135	13.246	−1.488	−16.737
2	3.227	7.309	−8.749	−1.707
3	−3.145	4.292	0.599	−1.746
4	−4.310	2.903	5.284	−3.878
5	5.258	−0.873	5.499	−9.470

When we consider each day as a time series within the same group we are implicitly assuming that each day of the week behaves similarly. This may not be the case for individuals and prior analysis of this data indicated there could be day of the week effects in some individuals [8]. To assess this, we conducted the same analysis but creating each time series from a week of data instead of a day. The method is agnostic to this choice and we simply modify the penalty to be 8log(96×7) to reflect the dimension change.

Figure 7 depicts the weekly changepoints there are at least three, usually four, changepoints each day. The changepoints in the weekly analysis are very similar to the daily analysis. The first changepoint (07:30 a.m.) is identified either exactly (3 days) or within 30 min (4 days). Similarly, the final changepoint (10:15 p.m.) is identified exactly (4 days) or within 15 min (3 days). The changepoint at 5:30 p.m. is only identified exactly on Tuesday and at 6:00 p.m. on Thursday. Tables 6 and 7 give the global mean probability of activity and the random contribution to the probability of events occurring in each segment. These show that, while there is some variability in the changepoints across days, the morning wake‐up and evening wind‐down times are fairly consistent across days of the week, and the variability is in the probability of activity within a day. These findings are important for setting alerts to family members or carers if an individual has not triggered a sensor in the morning, or is continuing to trigger sensors past their typical sleep time. In contrast, warnings within a day are likely to be less useful as the routine across days can vary considerably more.

**FIGURE 7:**
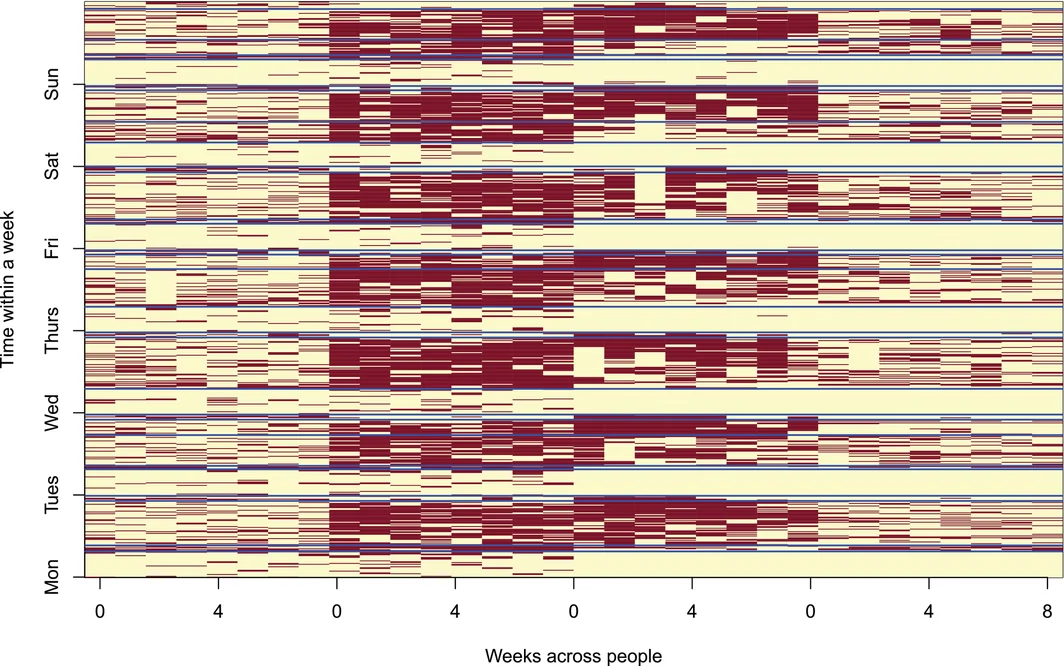
Weekly Howz data with changepoints. The x‐axis shows weeks for each person, across four people.

**TABLE 6:** Probability of activity and their associated 95% bootstrap confidence intervals across segments in Howz weekly analysis.

Segment	1	2	3	4	5	6	7	8	9	10
2.5% level	0.000	0.349	0.238	0.062	0.000	0.108	0.257	0.182	0.044	0.000
Mean	0.005	0.600	0.469	0.221	0.004	0.435	0.457	0.511	0.224	0.005
97.5% level	0.178	0.825	0.713	0.526	0.192	0.817	0.670	0.825	0.604	0.120

Segment	11	12	13	14	15	16	17	18	19	20
2.5% level	0.272	0.057	0.000	0.245	0.220	0.016	0.000	0.027	0.261	0.012
Mean	0.504	0.213	0.017	0.458	0.517	0.200	0.015	0.463	0.465	0.148
95.5% level	0.739	0.547	0.159	0.662	0.830	0.692	0.116	0.971	0.677	0.535

Segment	21	22	23	24	25	26	27	28	29
2.5% level	0.002	0.254	0.274	0.000	0.004	0.000	0.239	0.292	0.008
Mean	0.019	0.457	0.533	0.266	0.034	0.158	0.442	0.554	0.086
97.5% level	0.110	0.675	0.795	0.874	0.154	0.887	0.663	0.802	0.392

**TABLE 7:** Contribution of the random effects, on the logit scale, of activity across segments and people in Howz weekly analysis.

Person segment	1	2	3	4
1	3.286	4.019	−2.065	−2.065
2	0.097	1.491	−1.311	−0.316
3	−1.080	0.992	1.003	−0.913
4	0.883	−0.192	1.366	−1.871
5	3.201	4.041	−2.051	−2.051
6	1.631	1.297	−2.570	−0.239
7	−1.051	1.222	0.251	−0.419
8	−1.122	0.641	2.041	−1.568
9	1.369	−1.306	1.786	−1.620
10	2.952	3.775	−1.964	−1.964
11	−0.905	1.511	0.199	−0.807
12	1.008	−1.195	1.570	−1.195
13	1.666	2.758	−1.205	−2.310
14	−0.757	1.461	−0.026	−0.676
15	−1.621	0.728	2.029	−1.145
16	1.457	−0.334	2.181	−2.822
17	1.753	2.458	−1.154	−2.207
18	0.443	3.389	−3.123	−0.688
19	−1.106	1.268	0.354	−0.514
20	1.864	−0.184	1.441	−2.676
21	1.116	2.152	−0.682	−2.004
22	−0.328	1.515	−0.791	−0.393
23	−1.322	1.083	1.200	−0.963
24	1.104	−0.362	3.392	−3.525
25	0.594	1.773	0.362	−2.358
26	2.614	3.785	−3.308	−1.846
27	−0.726	1.381	−0.794	0.145
28	−1.226	0.743	1.363	−0.885
29	1.612	−0.595	1.750	−2.254

### 4.2 Dynamic Functional Connectivity in Resting‐State fMRI

Functional connectivity (FC) is the area of neuroimaging research that studies patterns of interregional interactions in the brain. Resting‐state functional magnetic resonance imaging (rs‐fMRI) data, which consists of time series data across spatial units (e.g., voxels, parcels, and regions), can be used to noninvasively quantify FC. Static FC assumes stationarity in the time series data, and the simplest approach is to calculate the correlation matrix from the multivariate time series. However, there has been a rapid growth in interest in *dynamic* FC (dFC), which assumes that FC changes over time [28]. There are several complementary analytic pipelines for estimating dFC, with each approach making different assumptions about the data. Sliding window analyses [29] and hidden Markov models [30, 31, 32] are popular approaches for dFC analysis.

Our motivating data is from the Brain Imaging Development of Girls' Emotion and Self (BRIDGES) Study, a longitudinal study whose aim is to understand brain development in adolescents with non‐suicidal self‐injury (NSSI). The BRIDGES Study recruited individuals 12–16 years of age who identified as female at birth and who exhibited a continuum of NSSI severity representing four categories: No NSSI, Mild NSSI, Moderate NSSI, and Severe NSSI [33]. We will focus on a cross‐sectional assessment of the rs‐fMRI data from N=137 adolescents. Detailed descriptions of data acquisition and preprocessing has been reported elsewhere [34, 35]. Our overall goals in our analysis are to (i) carry out a dFC analysis to obtain each individual's number of changepoints, and then (ii) determine how dFC differs across NSSI severity.

Following our recent work demonstrating the relevance of the default network to suicidal risk in children [36], here we focused our analyses on brain regions from the core hubs of the default mode network, consisting of the anterior cingulate cortex (ACC), medial prefrontal cortex (mPFC), and posterior cingulate cortex (PCC). We used the Glasser atlas from the Human Connectome Project [37] and applied Glasser Clusters 18 and 19, which contain the PCC and ACC/mPFC, respectively. We used these two Clusters from the left and right hemispheres of the brain to form K=4 groups, with each group containing 14 parcels, leading to time series data from $p=\sum_{i=1}^{4}k_{i}=56$ spatial locations. We demeaned each time series, and removed the autocorrelation by fitting an autoregressive model of order 8 to each time series and extracted the residuals. This prewhitening approach is the same one advocated for by previous research for FC analyses [38, 39] and dFC analyses [40]. In addition to prewhitening being common practice for analyzing neuroimaging data, our simulations showed inflated false positives in the presence of changepoints when autocorrelation was present. We then scaled the residual time series to have unit variance, and we use the resulting time series in our analyses. In summary, our time series data had length n=904, and p=56 dimensions that formed K=4 groups with $k_{i}=14$ for each i=1,…,4, obtained from N=137 individuals. These individuals had NSSI classifications as follows: 40 No NSSI, 13 Mild NSSI, 51 Moderate NSSI, and 33 Severe NSSI.

For each individual, we fit the PELT‐LMEC‐UB and PELT‐LMEC‐HB methods to their data and then we extracted the number of changepoints obtained from each method. PELT‐LMEC‐UB detected 0 changepoints in 108 individuals. This could be due to the method being prone to false negatives, as shown in our simulations. PELT‐LMEC‐HB, on the other hand, detected a range of 0–5 changepoints across all individuals. Note that our approach does not presuppose that the time series is nonstationary. Indeed, we have evidence of nonstationarity in an individual's time series data when 1 or more changepoints were detected. Individuals exhibiting a higher number of changepoints demonstrate greater temporal dynamics within the default mode network.

Figure 8 shows the results from one individual for which PELT‐LMEC‐HB detected 2 changepoints, giving rise to three segments and hence three covariance matrices, and we converted each to a correlation matrix. These three correlation matrices across the three segments captures the temporal dynamics of functional connectivity between and within these brain regions for this specific individual. We can also see in Figure 8 the “static” functional connectivity for this individual, which is the correlation matrix of the data for this person assuming no changepoints. In contrast to static FC, dFC captures the changes in the correlations over time, for instance, stronger correlations were present during Segment 2 and weaker correlations were present during Segment 3. In addition, Figure 8 gives us a qualitative look at the block structure in the correlation matrices, both when assuming no changepoints and also within each segment after estimating the changepoints. The subregions within the PCC, in particular, are strongly inter‐correlated, and the PCC also shows strong between‐hemisphere correlations. In contrast, the ACC/mPFC has greater variability of the strength in the correlations between its subregions, and its between‐hemisphere block structure is not as strong as the PCC. Not shown in the figure are the variances of each dimension of the time series. For Segment 1, the variances for the ACC/mPFC Left, PCC Left, ACC/mPFC Right, and PCC Right, had ranges (0.84, 1.22), (0.84, 1.08), (0.81, 1.22), and (0.92, 1.15), respectively, thereby providing justification for the heterogeneous block assumption.

**FIGURE 8:**
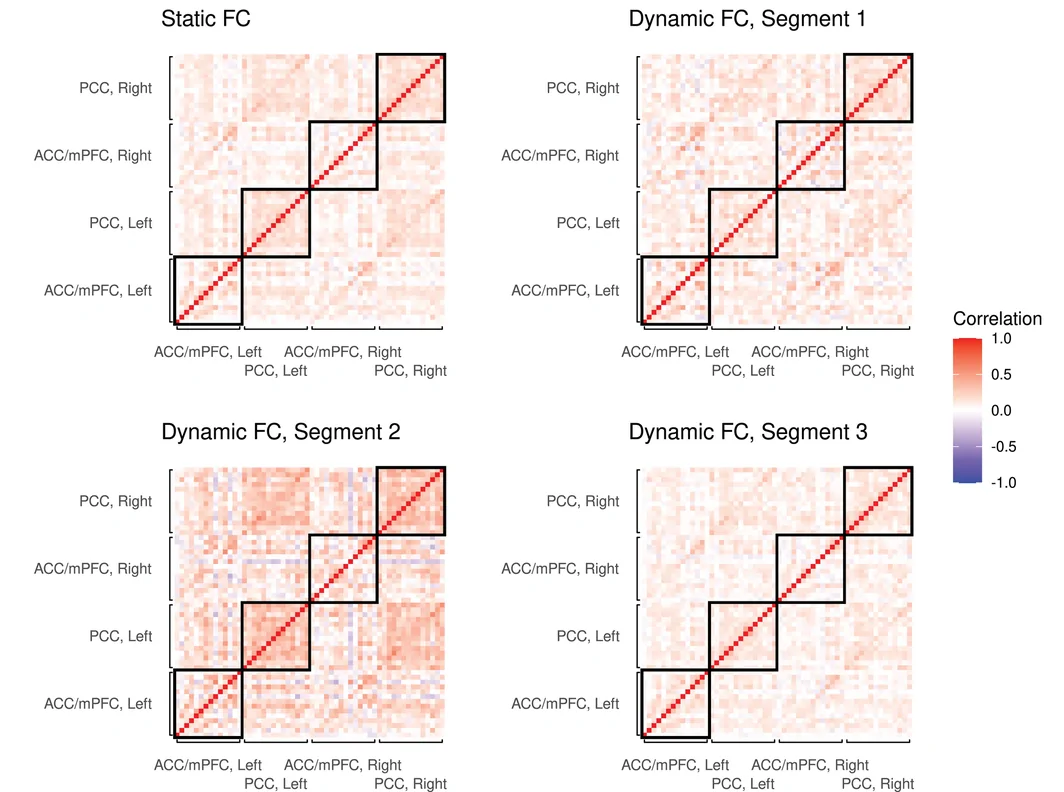
Results of the changepoint analysis using PELT‐LMEC‐HB from one individual. Two changepoints were detected, leading to three segments. The four matrices are correlation matrices, assuming no changepoints (Static FC) and also for each of the three segments (Dynamic FC). The black squares correspond to the regions of the brain: The anterior cingulate cortex (ACC) and medial prefrontal cortex (mPFC), and posterior cingulate cortex (PCC), for each of the left and right hemispheres. These four regions form the four groups in the analysis.

To determine if this measure of dFC has clinical relevance in this sample, we carried out a posthoc analysis using Poisson regression to determine the association between NSSI severity and the number of changepoints. We set the number of changepoints as the outcome and NSSI severity group as the predictor, with No NSSI as the reference group. Results from the Poisson regression indicated a higher mean for the number of changepoints for those with Mild, Moderate, and Severe NSSI relative to those with No NSSI, and the increase in the number of changepoints for both Moderate and Severe NSSI relative to No NSSI was statistically significant (*p*‐values = 0.017 and 0.023, respectively). Given recent literature on the impact of motion on dFC analyses [41], we also calculated each individual's framewise displacement (FD), which is a measure of motion at each time point; greater values of FD correspond to greater motion. We counted the number of time points that each individual's FD exceeded 0.3 mm and included this number as a regressor in the Poisson regression. After adjusting for FD and age, the direction of the effects was the same, but only the increase in the number of changepoints for those with Severe NSSI relative to No NSSI was statistically significant (*p*‐value = 0.033). Altogether, these results suggest greater temporal dynamics in the functional connectivity within the default mode network as NSSI severity worsens.

Our analysis showed that the connectivity within the default mode network had more changes over time, that is, greater temporal variability, among those with severe NSSI symptoms. This is consistent with previous works on the increased variability in the temporal dynamics of connectivity among those with depression or suicide ideation [42, 43, 44, 45]. There are, however, other works that reported a different direction of association [31, 46, 47]. These discrepancies can be attributed to the use of different modeling approaches for dFC (e.g., sliding‐window correlations, hidden Markov models, and graph theory), the analysis of data from different regions or networks of the brain, or a sample with vastly different characteristics (e.g., adults). We also point out that, while depression and NSSI highly overlap among youth [48], they are distinct processes with different biological and behavioral profiles [48, 49, 50, 51].

## 5 Conclusions

We used the mixed model framework to develop novel methods for changepoint analysis for time series data. The mixed model framework is flexible, which allowed us to model univariate time series from a potentially non‐normal distribution, or model the covariance matrix of multivariate time series. We embedded the mixed model in PELT for changepoint detection, giving rise to methods that are flexible with respect to data type and are computationally fast. Through extensive simulations, we showed the gains in performance of our methods relative to existing methods and showed how our methods perform in challenging scenarios. Finally, we illustrated the flexibility of our methods through two distinct applications.

There are parameters that must be set in advance, as described in Section 2.4, which will affect the method's performance. For instance, the combination of the minimum segment length and the penalty will limit the method's ability to detect changepoints. This can be critical for multivariate time series, particularly when the dimensionality of the data exceeds the length of the time series. A potential solution is to make strong assumptions about the data, for example, impose sparsity constraints in the covariance matrix. Our analysis focused only on the default mode network of the brain, and further developments in the method are needed to make it more scalable with respect to the dimensionality if whole‐brain analyses are of interest. Alongside assumptions on the cross‐correlation, we have assumed the time series themselves have zero autocorrelation. In our analysis of the neuroimaging data, we satisfied this assumption by prewhitening the time series and qualitatively inspecting the prewhitened data. Alternatively, the impact of autocorrelation can be mitigated either by dividing the penalty by the absolute autocorrelation to inflate the penalty or by explicitly modeling the autocorrelation, although this complicates the fitting of the models. Similarly, the changes that are identified are model‐based, so they are statistically meaningful but may not be clinically meaningful depending on the application. Post hoc investigations into, for example, the changepoint locations or how the parameters changed between segments can be helpful for interpreting the results.

Just as how PELT‐GLMER and PELT‐LMEC inherit the limitations of both the mixed modeling framework and PELT, they also inherit their strengths. The flexibility of the mixed model framework allows for natural extensions of our methods. We focused on changes in the mean (for PELT‐GLMER) or the covariance matrix (for PELT‐LMEC). Methods for estimating a precision matrix whenever the covariance matrix has a UB structure [12] may be adapted for the HB structure and into PELT‐LMEC. This would allow for changepoints in the graphical structure of the data, as explored in previous methods [9, 10]. We only used the UB and HB structures, but one can use other structures or make other assumptions (e.g., sparsity) for the covariance (or precision) matrix. However, one should be mindful of the number of parameters in the covariance matrix and the computational speed in estimating that structured covariance matrix.

## Funding

This work was supported by the National Institute of Mental Health (R01‐MH122473, R01‐MH107394); Engineering and Physical Sciences Research Council (EP/T021020/1, EP/T014105/1); Center for Magnetic Resonance Research (NIBIB P41 EB027061); High Performance Connectome Upgrade for Human 3 T MR Scanner (1S10OD017974‐01).

## Conflicts of Interest

The authors declare no conflicts of interest.

## Data Availability

The data that support the findings of this study are available from the corresponding author upon reasonable request.
